# Effects of concomitant obesity and diabetes on the aggressiveness and outcomes of differentiated thyroid cancer patients

**DOI:** 10.20945/2359-3997000000361

**Published:** 2021-04-27

**Authors:** Onur Elbasan, Dilek Gogas Yavuz

**Affiliations:** 1 Marmara University College of Medicine Division of Endocrinology and Metabolism Istanbul Turkey Division of Endocrinology and Metabolism, Marmara University College of Medicine, Istanbul, Turkey.

**Keywords:** Differantiated thyroid cancer, BMI, obesity, prediabetes, T2DM, metastasis

## Abstract

**Objective:**

Obesity and diabetes are the risk factors for cancer development including differentiated thyroid cancer (DTC). Contradictory accumulated data indicates the possible negative effects of obesity and hyperglyceamia as a factor for aggressiveness of DTC. The aim of the present study is to investigate the association of high body mass index (BMI) and presence of type 2 diabetes mellitus (T2DM) on the histological aggressiveness and clinical outcomes in DTC patients followed for over 4 years in a single center.

**Materials and methods:**

Consequative 526 DTC patients who had undergone total thyroidectomy and/or radioactive iodine (RAI) ablation were reviewed retrospectively. Patients were divided into groups based on their BMI: normal weight, overweight, obese and also were evalauted in 3 groups presence of diabetes, prediabetes and nomoglyceamia. Histological aggressiveness of DTC at the time of diagnosis and clinical response at the time of last clinical visit were reassessed according to the criteria suggested by ATA 2015 guideline.

**Results:**

No differences in histopathologic features, risk of recurrence, cumulative dose of RAI ablation and prevalence of 131I avid metastatic disease were demonstrated among the groups both classified according to BMI and hyperglycemia. Mean of 3.4 year follow-up also showed no differences in the clinial repsonse to therapy and percentage of nonthyroid primary cancer in DTC patients.

**Conclusion:**

In this retrospective study we demonstrated that obesity and T2DM have no additive effect on DTC aggressiveness and response to therapy. DTC patients with obesity and diabetes can be treated according to present guidelines without requirement for spesific attention.

## INTRODUCTION

Thyroid cancer is one of the most common endocrine malignancies and its incidence rate has increased significantly in the last decades. According to 2016 standardized data in Turkey, the frequency of thyroid carcinoma was 62-229 per 1,000,000 persons ( 1 ). Although increased incidence of thyroid carcinoma is attributed to rising awareness and easier diagnosis, accumulating data have suggested that concomitant diseases such as diabetes and obesity may play a role ( 2 – 5 ). Obesity and type 2 diabetes mellitus (T2DM) are complex metabolic disorders, and epidemiological studies have indicated their association with increased risks for several cancers including colon, breast, pancreas, liver, endometrial and thyroid ( 6 , 7 ). The possible mechanisms with increased thyroid cancer risk in obesity and diabetes are still unclear. Hyperinsulinemia, insulin resistance and proinflamatory state may have an effect on thyroid carcinogenesis through insulin receptors overexpressed in cancer cells ( 4 , 5 , 8 ). According to different studies, obese and diabetic patients may have an increased risk of malignancy and differentiated thyroid cancer (DTC) aggressiveness as a result of clinically higher serum thyroid stimulating hormone (TSH) levels compared to the normal population ( 9 – 11 ).

Body mass index (BMI) has been linked to a higher incidence of thyroid cancer in some cohorts ( 2 , 3 ). Some studies have found that obesity is associated with more advanced stage or aggressive cancers at presentation and the recurrence or metastasis for several types of cancers, but it has not been confirmed with recent studies for thyroid cancer ( 12 – 15 ). Along with the studies indicating an increased risk of thyroid cancer in diabetic patients ( 4 , 5 ), there are also studies showing the lack of association ( 16 – 18 ). Retrospective and prospective clinical studies reported conflicting results and the effects of obesity and diabetes on the clinical outcomes of thyroid cancer have not yet been clarified. In the present study, we aimed to investigated the associations of BMI, prediabetes and T2DM with pathological features and clinical outcomes of DTC patients followed for over 4 years in a single center.

## MATERIALS AND METHODS

The Study Protocol was approved by the ethics committee of Marmara University School of Medicine (protocol number: 09.2018.494). The study was conducted in accordance with the Declaration of Helsinki. In this retrospective study consequtive 526 DTC patients followed-up from January 2010 to December 2018 were included in the study. Patients who had undergone total thyroidectomy and diagnosed with papillary, follicular and hurtle cell thyroid carcioma through pathologic examination of the surgical specimen were enrolled. Demographic parameters, histopathological findings, surgical reports, radioactive iodine ablation history, radiologic evaluation reports after surgery (neck ultrasonography, iodine scans), clinical data [duration of disease, follow-up time, daily dose of levothyroxine, history of obesity, prediabetes and T2DM, weight, height, body mass index (BMI:calculated by weight/height^2^)] were recorded. Avaible biochemical data of fasting plasma glucose (FPG), HbA1c levels at the time of diagnosis, and thyroid stimulating hormone (TSH), free thyroxine (T4), thyroglobulin (Tg) and thyroglobulin antibody (TgAb) values after surgery and also at the last visit were recorded. Patients were categorized in three groups according to their BMI: normal weight (18.5-24.9 kg/m^2^), overweight (25-29.9 kg/m^2^) and obese (BMI ≥ 30 kg/m^2^), and were also classified in three groups according to their glycemic status considering diabetes history, FPG, Hba1c values as normoglycemia (FPG < 100 mg/dL and/or HbA1c < 5.7), prediabetes (FPG = 100-125 mg/dL and/or HbA1c = 5.7%-6.4%) and diabetes (FPG ≥ 126 and/or HbA1c ≥ 6.5) according to the ADA 2019 guideline. The clinicopathological findings were compared in the groups were categorized according to BMI and glycemic disorders. As a routine procedure, patients were periodically followed-up at regular intervals with biochemical and ultrasonographic evaluations, and the follow-up interval and other diagnosis and/or treatment options were determined according to the dynamic risk reclassification process ( 19 ). Thyroglobulin values were taken into account only in the absence of thyroglobulin antibodies (TgAb) to elicit clinical response. Changes in levels over time were accepted to describe the response to treatment in the presence of TgAb ( 20 ). Development of new metastatis after surgical removal, RAI ablation requirement, cumulative RAI doses, nonthyroid primary cancer were recorded for the whole follow up period.

### Risk evaluation at the time of diagnosis

Stage and risk of metastasis/recurrence were evaluated according to the American Joint Committee on Cancer (AJCC) 7^th^ Edition Staging System for Differentiated Thyroid Carcinoma using the AMES (Age, Metastases, Extent, Sex) score at the time of diagnosis ( 21 ). We also stratified the risk of recurrence based on the ATA 2009 guideline as Low, Intermediate and High categories ( 19 ).

### Radioactive iodine ablation treatment

Thyroid remnant ablation was performed 4-6 weeks after surgery. The 131I ablation dose for almost all patients was 100 mCi (millicuries). A whole body scan (WBS) was performed 1 week after 131I administration. The first 131I treatment was conducted after recombinant human TSH stimulation (rhTSH) in 70.3% of patients (n:370) and after 3-4 weeks of withdrawal of L-thyroxinere placement therapy in 29.7% of patients (n:156). TSH stimulation was obtained by administering 1 injection of rhTSH on 2 consecutive days. 131I neck take-up was measured for diagnostic purposes at 3 and 24 hours after the administration of 0.05 mCi of 131I. Based on the radioiodine enhancement, we distinguished the post-therapeutic WBS results as negative; when the presence of 131I uptake was exclusively present in the thyroid remnant or positive; for the presence of uptake related to lymph nodes and/or distant metastases. WBS requirement in follow-up was determined according to risk reassessment.

### Reassesment of clinical response

The clinical response was assessed at the time of the last outpatient clinic visit, according to the criteria suggested by the 2015 ATA guideline with evaluation of postoperative Tg and imaging methods ( 22 ). The patients were evaluated according to the final response to therapy as excellent, biochemical incomplete, structural incomplete and indeterminate.

### Biochemical Parameters

Serum TSH (N: 0.34-5.6 μIU/mL), free T4 (N: 0.61-1.12 ng/dL), Tg and TgAb was measured in automated serum samples by paramagnetic particle chemiluminescence immunoassay method (DxI800, Beckman Coulter, USA). Serum glucose levels were automatically measured by the hexokinase method (AU5800, Beckman Coulter, USA). HbA1c percentages were measured in EDTA whole blood samples by boranetafinite chromatography (TrinityBiotech, Ireland). Tg ≤ 0.2 andTgAb ≤ 0.9 values were considered as remission.

### Statistical analysis

The distribution of the data was analysed by Shapiro-Wilk Test. One-Way Anova test was used for comparing the data showing normal distribution among three groups. The comparing of the variables that do not have normal distribution among three groups was analysed by Kruskal-Wallis ANOVA Test. Fisher Exact Test were applied for analysing the differences between categoric data. Post-hoc comparings of the variables that were found meaningful after Anova and Kruskal Wallis tests were analysed by Dunn test. We gave frequency for the following descriptive statistics of data for numerical variables: average, standard deviation, median, minimum, and categorical variables. All analyses were made by IBM Statistics 22.0 Program within the 0.05 significance level.

## RESULTS

The epidemiological and clinicopathological features of study population are shown in Table 1 . Of 526 DTC cases involved in the study, 512 (97.34%) were papillary thyroid carcinoma, 12 (2.28%) were follicular and 2 (0.38%) were Hurthle cell carcinoma. The median age at evaluation was 51 years and the patients were predominantly female (81%). The mean BMI was 30.55 ± 4.88 kg/m^2^; 53% of the cases were obese, 37.3% were overweight and 9.7% were normal weight. T2DM frequency was 19.6%, 31.7% of the patients were prediabetic and 48.7% were normoglycemic. The rate of metastasis was 18.1% and 52.1% of the patients were at high risk according to the AMES score. The risk of recurrence was found to be low in more patients (63.9%) according to ATA, and 65.2% of the patients were classified as Stage 1 based on the TNM system.

**Table 1:** Clinical, histopathologic and loboratory characteristics of DTC patients

Parameter		N (%)
Age (years)		51 (19-88)
Gender (female/male)		426 (81%)/100 (19%)
Age at diagnosis (years)		44.55 ± 12.65
Duration of disease (years)		4 (1-37)
Follow-up time (years)		3.4 ± 2.58
Daily dose of LT4 (mcg)		119.63 (25-300)
LT4 dose per kilogram (mcg/kg)		1.62 ± 0.52
Cumulative RAI dose (mCi)		100 (30-600)
Nonthyroid primary cancer		44 (8.4%)
Histology		
	Papillary carcinoma	512 (97.34%)
	Follicular carcinoma	12 (2.28%)
	Hurtle cell carcinoma	2 (0.38%)
Body mass ındex (kg/m^2^)		30.55 ± 4.88
	Obese/overweight/normal weight	279 (53%)/196 (37.3%)/51 (9.7%)
Glycemic Status		
	T2DM/prediyabetes/normoglycemia	103 (19.6%)/167 (31.7%)/256 (48.7%)
Pathology		
Tumor size (mm)		11 (4-110)
Multicentric tumor (n)		159 (30.23%)
Lymphatic invasion (n)		67 (12.7%)
Vascular invasion (n)		75 (14.2%)
Extrathyroidal soft tissue invasion		99 (18.8%)
Metastasis		95 (18.1%)
	Cervical lymph node metastasis	81 (15.4%)
	Distant metastasis	14 (2.7%)
Laboratory		
	TSH (μIU/mL)	0.4 (0.001-121)
	Free T4 (ng/dL)	1.07 (0.06-5.15)
	Thyroglobuline (ng/mL)	0.2 (0.04-4154)
AMES risk (AJCC 7th edition)		
	High	274 (52.1%)
	Low	252 (47.9%)
Risk of recurrence (ATA)		
	High	24 (4.6%)
	Intermediate	166 (31.5%)
	Low	336 (63.9%)
Staging		
	I	343 (65.2%)
	II	13 (2.5%)
	III	137 (26%)
	IV	33 (6.3%)
Response to therapy		
	Excellent	239 (45.5%)
	Biochemical incomplete	36 (6.8%)
	Sutructural incomplete	17 (3.2%)
	Indetermine	234 (44.5%)

Clinical and demographic features according to BMI are presented in Table 2 . Obese and overweight groups have a higher age than the overweight and normal weight groups (p = 0.003). The age at diagnosis in the obese group was also significantly higher than the other groups (p = 0.017). There was no difference between the groups in terms of histopathological features such as tumor size, multicentricity, lymphatic, vascular and extrathyroidal invasion and laboratory parameters such as TSH, free T4 and Tg. There was no significant difference between the groups in terms of metastasis, AMES and ATA high risk patient ratio. The rate of Stage 1 cases and patients with excellent response to therapy was also similar in groups. Cumulative RAI dose and nonthyroid primary cancer rates did not differ between BMI groups.

**Table 2:** Clinical, laboratory and histopathologic evalauation of DTC patients according to BMI

	Normal weight (n = 51)	Overweight (n = 196)	Obese (n = 279)	p
Age (years)	46.18 ± 13.7	49.76 ± 13.11	52.18 ± 11.98	**0.003**
Female (n)	38 (74.51%)	141 (71.94%)	247 (88.53%)	**<0.001**
Age at DTC diagnosis (years)	40.86 ± 13.76	43.67 ± 13.18	45.84 ± 11.9	**0.017**
Duration of disease (years)	4 (1-17)	3 (1-37)	4 (1-30)	0.415
Daily dose of LT4 (mcg)	103.57 (57.14-200)	116.06 (50-235.71)	125 (25-300)	**0.023**
Tumor size (mm)	11 (1-85)	11.5 (1-80)	11 (0.4-110)	0.821
Multicentric tumor (n)	13 (25.49%)	68 (34.69%)	78 (27.96%)	0.215
Lymphatic invasion (n)	10 (19.61%)	28 (14.28%)	32 (11.47%)	0.297
Vascular invasion (n)	9 (17.65%)	28 (14.28%)	38 (13.62%)	0.796
Extrathyroidal soft tissue invasion (n)	10 (19.61%)	38 (19.39%)	51 (18.28%)	0.894
All metastatis	12 (23.53%)	41 (20.92%)	42 (15.05%)	0.148
Cervical lymph node metastasis	11 (21.57%)	34 (17.35%)	37 (13.26%)	0.525
TSH (μIU/mL)	0.83 (0.01-45.98)	0.28 (0.001-121)	0.47 (0.01-100)	0.069
Free T4 (ng/dL)	1.04 (0.41-1.76)	1.1 (0.06-3.06)	1.05 (0.16-5.15)	0.321
Tg (ng/mL)	0.2 (0.2-25.2)	0.2 (0.04-2475)	0.2 (0.04-4154)	0.053
AMES high risk	34 (66.67%)	102 (52.04%)	138 (49.46%)	0.104
High risk of recurrence (ATA)	1 (1.96%)	11 (5.61%)	12 (4.3%)	0.791
Stage 1	33 (64.7%)	133 (67.86%)	177 (63.44%)	0.350
Excellent response to therapy	20 (39.42%)	92 (46.94%)	127 (45.52%)	0.880
Cumulative RAI dose (mCi)	100 (50-400)	100 (30-600)	100 (30-400)	0.916
Nonthyroid primary cancer	4 (7.84%)	17 (8.67%)	23 (8.24%)	0.976

DTC: differentiated thyroid cancer; LT4: L-thyroxine; TSH: thyroid stimulating hormone; T4: thyroxine; Tg: thyroglobuline; ATA: American Thyroid Association; AJCC: American Joint Committee on Cancer; AMES: age, metastases, extent, sex; RAI: radioactive iodine ablation.

Clinicopathological features among three groups according to glycemic status are demonstrated in Table 3 . The mean age and age at DTC diagnosis of the diabetic patients were significantly higher than other groups (p < 0.001, p < 0.001). The female gender ratio and BMI were found to be significantly higher in the prediabetic group compared to the other groups (p = 0.043, p < 0.001, respectively). Tumor diameter did not differ between groups. There was also no significant difference between the groups in terms of multicentricity, tumor invasion parameters and metastasis characteristics. Also, TSH, free T4 and Tg levels were not significantly different between groups. There was no significant difference between the groups regarding the rate of patients with AMES and ATA high risk, Stage 1, and excellent response to treatment. Cumulative RAI doses and the rate of nonthyroid primary cancer were similar in all groups.

**Table 3:** Clinical, laboratory and histopathologic evaluation of DTC patients according to glycemic status

	Normoglycemia (n = 256)	Prediabetes (n = 167)	Diabetes (n = 103)	p
Age (years)	46.25 ± 13.02	52.6 ± 10.56	59.3 ± 9.89	**<0.001**
Female(n)	209 (81.64%)	142 (85.03%)	75 (72.82%)	**0.043**
BMI (kg/m^2^)	29.3 (17.3-53.67)	31.63 (18.73-58.25)	31.24 (17.72-47.42)	**<0.001**
HbA1c (%)	5.3 (4.2-5.6)	5.9 (4.4-6.4)	6.5 (5.0-13.4)	**<0.001**
Age at DTC diagnosis (years)	39 (13-76)	46 (23-78)	53 (8-78)	**<0.001**
Duration of DTC (years)	3 (1-29)	5 (1-30)	4 (1-37)	**0.006**
Daily dose of LT4 (mcg)	121.42 (25-300)	114.28 (50-271.42)	128.57 (25-300)	0.082
Tumor size (mm)	12 (1-110)	12 (1-80)	10 (0.4-70)	0.05
Multicentric tumor (n)	88 (34.38%)	44 (28.35%)	27 (26.21%)	0.131
Lymphatic invasion (n)	36 (14.06%)	19 (11.38%)	12 (11.65%)	0.418
Vascular invasion (n)	41 (16.02%)	23 (13.77%)	7 (6.8%)	0.066
Extrathyroidal softtissue invasion (n)	50 (19.53%)	32 (19.16%)	17 (16.5%)	0.502
All metastatis	51 (19.92%)	24 (14.37%)	20 (19.42%)	0.322
Cervical lymph node metastasis	43 (16.8%)	22 (13.17%)	17 (16.5%)	0.930
TSH (μIU/mL)	0.35 (0.001-56.04)	0.4 (0.01-121)	0.44 (0.02-59)	0.267
Free T4 (ng/dL)	1.08 (0.25-3.59)	1.06 (0.06-5.15)	1.07 (0.16-4.58)	0.698
Tg (ng/mL)	0.2 (0.04-2475)	0.2 (0.04-558)	0.2 (0.04-4154)	0.259
AMES high risk	140 (54.69%)	89 (53.29%)	45 (43.69%)	0.157
High risk of recurrence (ATA)	12 (4.69%)	8 (4.79%)	4 (3.88%)	0.952
Stage 1	181 (70.7%)	104 (62.28%)	58 (56.31%)	0.087
Excellent response to therapy	118 (46.09%)	73 (43.71%)	48 (46.6%)	0.150
Cumulative RAI dose (mCi)	100 (30-600)	100 (50-300)	100 (30-600)	0.823
Nonthyroid primary cancer	17 (6.64%)	18 (10.78%)	9 (8.74%)	0.320

BMI: body mass index; DTC: differentiated thyroid cancer; LT4: L-thyroxine; TSH: thyroid stimulating hormone; T4: thyroxine; Tg: thyroglobuline; ATA: American Thyroid Association; AJCC: American Joint Committee on Cancer; AMES: age, metastases, extent, sex; RAI:r adioactive iodine ablation.

## DISCUSSION

In this retrospective study, we observed no additive effect of the presence of obesity and T2DM on the histological and clinical aggressiveness of DTC patients followed in a single center. This result is concordant with numerous studies in the literature, although a few clinical studies have reported contrary results. In a study conducted in a large group of patients, there was no significant relationship between BMI and tumor size, multifocality, extrathyroidal invasion, cervical lymph node metastasis or distant metastasis ( 13 ). In a retrospective study, Paes and cols. reported a lack of association between BMI and histological aggressiveness of DTC features ( 12 ). Similarly, in a Polish study including 1181 patients, BMI was not found to be a risk factor for the aggressiveness of DTC ( 15 ). A Korean series further showed that obesity influenced larger tumor size, the presence of extrathyroidal invasion and advanced TNM stage ( 23 ). We did not show any histopathological and clinical difference between obese, overweight and normal weight patients in terms of tumor size, multicentricity, tumor invasion characteristics and metastasis ratio in accordance with the literature, as many clinical studies did not demonstrate tumor size and invasiveness according to BMI in DTC patients ( 12 , 13 , 24 ). In another research, patients were evaluated according to the ATA risk stratification system and significant relationship was not found between BMI and ATA risk score ( 14 ). In an Italian cohort, obese patients had less Stage I disease, compared to overweight and normal weight patients, but it was not statistically significant. In the same study, there was also no difference between the groups in terms of the response to therapy ( 24 ). Also in our study, the rate of Stage 1 disease was lower in the obese group, which was not statistically significant. Patients with AMES and ATA high risk score, and who had excellent response to treatment were also similar between obese, overweight and normal weight groups. In our study, the rate of overweight (37.3%) and obese (53%) patients is higher compared to the other studies mentioned above that examined the effect of obesity on thyroid cancer.

DTC aggressiveness was less invastigated in diabetic and prediabetic patients. A prospective study indicated that the worse pathological features were higher in diabetic patients than nondiabetics ( 25 ). However, in a study including 8-year follow-up ( 26 ), no significant difference was observed between the diabetic and control groups in terms of clinicopathological characteristics. In a retrospective study, it was mentioned that T2DM was associated with the advanced TNM stage in DTC cases ( 27 ), whereas another study comparing diabetic and nondiabetic patients by matching them exactly did not find a difference regarding clinical stages ( 25 ). Although the rate of stage 1 disease was lower in the prediabetic and diabetic groups compared to the normoglycemic group in our study, we did not observe a significant difference. The risk of recurrence based on ATA and AMES scores was also similar in all groups ( Table 3 ). We could not find any difference of DTC cancer aggressiveness in diabetic and prediabetic patients compared to normoglycemic ones. We can state that the low median HbA1c values (6.5%) of the diabetic patients in our study, that is the higher rate of controlled diabetes compared to other studies mention above, may contribute to this result.

Metastasis in DTC was also previously evaluated in diabetic and nondiabetic patients, and T2DM has been shown to rise the metastasis ( 25 , 27 ). In contrast, no significant difference was found between the normoglycemic, prediabetic and diabetic groups in terms of metastasis and response to therapy in our study. This may be related to the generally low metastasis rate in this study compared to others. It can be considered the fact that the initial dose of RAI ablation was 100 mCi in almost all of our patients is effective on low metastasis rates, clinical outcome and response to therapy.

Since the increasing effects of obesity and T2DM on other types of cancer are known ( 27 ), we also examined other nonthyroidal cancers in our study and found that the rate of nonthyroid primary cancer was similar in all groups according to BMI and glycemic status.

In our research, the rate of obesity was 53% and the rate of T2DM was 19.6% and these rates are higher compared to our country and other studies ( 12 – 14 , 23 – 29 ). In spite of these high rates of diabetes and obesity, we have reported that they have no effect on histopathological characteristics and the outcome of thyroid cancer. The limitations of this study can be stated as followes: The retrospective structure, patient heterogeneity, not exactly knowing the duration of T2DM and prediabetes, the difficulty of separating and analyzing obesity and T2DM which are intertwined conditions, and the lack of clear knowledge of the effects of the drugs on weight and glycemic status.

In conclusion, according to our study results of Caucasian subjects followed-up in a single center, we demonstrated that the presence of obesity and diabetes have no additive effect on the aggressiveness and response to therapy in DTC patients. DTC patients with obesity and diabetes may be treated according to present guidelines with no specific attention requirement.
